# Clerodane Diterpenoids from *Callicarpa hypoleucophylla* and Their Anti-Inflammatory Activity

**DOI:** 10.3390/molecules25102288

**Published:** 2020-05-13

**Authors:** Yu-Chi Lin, Jue-Jun Lin, Shu-Rong Chen, Tsong-Long Hwang, Shu-Yen Fang, Michal Korinek, Ching-Yeu Chen, Yun-Sheng Lin, Tung-Ying Wu, Ming-Hong Yen, Chih-Hsin Wang, Yuan-Bin Cheng

**Affiliations:** 1 Graduate Institute of Natural Products, Center for Natural Product Research and Development, College of Pharmacy, Kaohsiung Medical University, Kaohsiung 80708, Taiwan; m8952612@hotmail.com (Y.-C.L.); conk4wu0g3@gmail.com (J.-J.L.); highshorter@hotmail.com (S.-R.C.); 2 Graduate Institute of Natural Products, Graduate Institute of Biomedical Sciences, College of Medicine, Chang Gung University, Taoyuan 33302, Taiwan; htl@mail.cgu.edu.tw (T.-L.H.); susan850903@gmail.com (S.-Y.F.); mickorinek@hotmail.com (M.K.); 3 Research Center for Industry of Human Ecology, Research Center for Chinese Herbal Medicine, and Graduate Institute of Health Industry Technology, College of Human Ecology, Chang Gung University of Science and Technology, Taoyuan 33302, Taiwan; 4 Department of Anesthesiology, Chang Gung Memorial Hospital, Taoyuan 33302, Taiwan; 5 Department of Physical Therapy, Tzu-Hui Institute of Technology, Pingtung 92641, Taiwan; chingyeu1971@yahoo.com.tw; 6 Department of Biological Science and Technology, Meiho University, Pingtung 912009, Taiwan; x00010106@meiho.edu.tw (Y.-S.L.); kuma0401@gmail.com (T.-Y.W.); 7 Department of Food Science and Nutrition, Meiho University, Pingtung 912009, Taiwan; 8 School of Pharmacy, College of Pharmacy, Kaohsiung Medical University, Kaohsiung 80708, Taiwan; yen@kmu.edu.tw; 9 Division of Pharmacy, Zuoying Branch of Kaohsiung Armed Forces General Hospital, Kaohsiung 81342, Taiwan; 10 Department of Marine Biotechnology and Resources, National Sun Yat-sen University, Kaohsiung 80424, Taiwan; 11 Department of Medical Research, Kaohsiung Medical University Hospital, Kaohsiung 80708, Taiwan

**Keywords:** *Callicarpa hypoleucophylla*, clerodane diterpenoid, anti-inflammatory activity

## Abstract

Plants of the genus *Callicarpa* are known to possess several medicinal effects. The constituents of the Taiwan endemic plant *Callicarpa hypoleucophylla* have never been studied. Therefore, *C. hypoleucophylla* was selected for our phytochemical investigation. Two new clerodane-type diterpenoids, named callihypolins A (**1**) and B (**2**), along with seven known compounds were isolated from the leaves and twigs of the Lamiaceae plant *C. hypoleucophylla* and then characterized. The structures of compounds **1** and **2** were elucidated by spectroscopic data analysis, specifically, two-dimension nuclear magnetic resonance (NMR). The anti-inflammatory activity of compounds **1**–**9** based on the suppression of superoxide anion generation and elastase release was evaluated. Among the isolates, compounds **2**–**4** showed anti-inflammatory activity (9.52−32.48% inhibition at the concentration 10 μm) by suppressing superoxide anion generation and elastase release. Our findings not only expand the description of the structural diversity of the compounds present in plants of the genus *Callicarpa* but also highlight the possibility of developing anti-inflammatory agents from *Callicarpa* endemic species.

## 1. Introduction

*Callicarpa* (family Lamiaceae) is a genus of about 190 species of herbaceous plants. The plant is geographically found throughout east and southeast Asia, Australia, Madagascar, southeast North America, and South America [1]. Folkloric usage of various parts of *Callicarpa* includes preparations used as fish poisons [2,3], insect repellents [1], and for some medical indications [3]. The phytochemical investigation of this genus has resulted in the identification of diterpenoids, phenylethanoids, phenypropanoids, and flavonoids. These components display various biological effects, such as anti-inflammatory [4,5,6], anti-platelet aggregation [7], hemostatic [8], antioxidative [9,10], cytotoxic [6,11,12], and neuroprotective [13], antitubercular [14], hepatoprotective [15,16], antimicrobial [17], anti-arthritic [18], as well as analgesic properties [19]. From the above-mentioned phytochemical and biological studies, we know this genus may offer a rich supply of bioactive phytochemicals. Because the phytochemical profile of the Taiwanese endemic plant *Callicarpa hypoleucophylla* has never been analyzed, we carried out an investigation of the constituents and bioactivity of *C. hypoleucophylla*. A meticulous separation of an ethanolic extract of *C. hypoleucophylla* led to the isolation of two new clerodane-type diterpenoids that we named callihypolins A and B (**1** and **2**), together with seven known analogues (**3**–**9**). The anti-inflammatory evaluation of these isolates is also presented in this paper.

## 2. Results and Discussion

**Figure 1:**
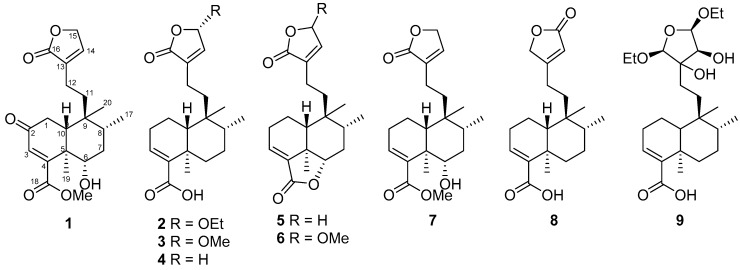
Structures of compounds **1**–**9** isolated from *Callicarpa hypoleucophylla*.

The leaves and twigs of *C. hypoleucophylla* were extracted with 95% ethanol; the yielded extracts were suspended in H_2_O and extracted with ethyl acetate (EtOAc). The EtOAc-soluble part was further partitioned with hexanes/methanol (MeOH)/H_2_O (4:3:1) to obtain a MeOH layer. The MeOH layer was subjected to extensive chromatography by normal- and reversed-phase HPLC, using a normal-phase silica gel open column and a Sephadex LH-20 resin column, supplying callihypolins A and B (**1** and **2**) as well as seven known compounds (4a*R*,5*S*,6*R*,8a*R*)-5-[2-(2,5-dihydro-5-methoxy-2-oxofuran-3-yl)ethyl]-3,4,4a,5,6,7,8,8a-octahydro-5,6,8a-trimethylnaphthalene-1-carboxylic acid (**3**) [20], patagonic acid (**4**) [21], limbatolide F (**5**) [22], limbatolide A (**6**) [23], methyl (4a*R*,5*S*,6*R*,8*S*,8a*R*)-3,4,4a,5,6,7,8,8a-octahydro-8-hydroxy-5,6,8a-trimethyl-5-[2-(2-oxo-2,5-dihydrofuran-3-yl)ethyl]naphthalene-1-carboxylate (**7**) [24], clerodermic acid (**8**) [25], and visclerodol acid (**9**) [26] (Figure 1).

**Figure 2:**
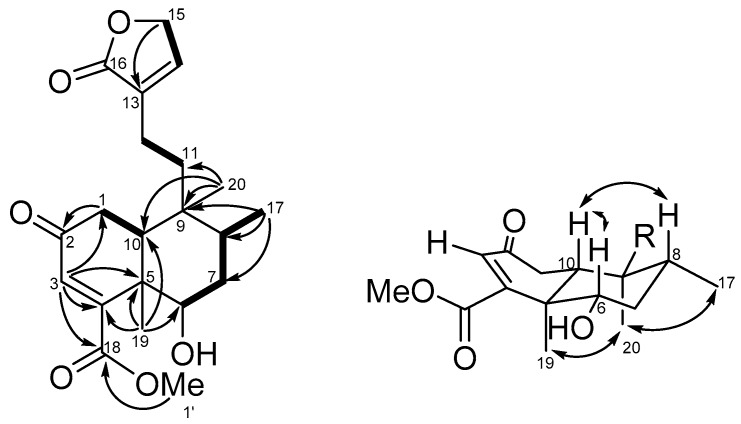
COSY (bold bond), selected HMBC (arrow), and NOESY (left-right arrow) correlations of compound **1**.

The molecular formula of compound **1** was established to be C_21_H_28_O_6_ on the basis of the [M + Na]^+^ peak at *m/z* 399.17785 (calcd. 399.17781 for C_21_H_28_O_6_Na) obtained from high-resolution electrospray ionization mass spectrometry (HRESIMS) (Figure S13). The IR absorption bands of compound **1** indicated the presence of hydroxy (3451 cm^−1^), α,β-unsaturated-γ-lactone (1739 cm^−1^), and carboxyl (1678 cm^−1^) functionalities. The ^13^C and distortionless enhancement by polarization transfer (DEPT)-135 NMR data (Figure S2) showed the presence of 21 carbons divided into 7 quaternary carbons (including 3 carbonyls), 5 methines, 5 methylenes, and 4 methyls. The ^1^H (Figure S1) and ^13^C NMR signals of compound 1 showed some characteristic peaks such as an olefinic methine singlet (δ_H_ 5.96, δ_C_ 126.6, C-3), two tertiary methyls (δ_H_ 1.33, δ_C_ 14.0, Me-19; δ_H_ 0.84, δ_C_ 17.3, Me-20), a secondary methyl (δ_H_ 0.90, *J* = 6.8 Hz, δ_C_ 15.3, Me-17), as well as a butenolide unit (δ_C_ 134.0, C-13; δ_H_ 7.09, 1 H, quin, *J* = 1.7 Hz, δ_C_ 143.9, C-14; δ_H_ 4.77, 1H, dd, *J* = 3.8, 1.7 Hz, δ_C_ 70.2, C-15; δ_C_ 174.0, C-16). The above NMR data indicated that the structure of compound **1** was similar to that of dichrocephnoid E [27], a clerodane diterpenoid, except for a methylene corresponding to C-6 that was replaced by an oxymethine (δ_H_ 3.84, δ_C_ 72.5) and an additional methoxy (δ_H_ 3.81, δ_C_ 52.8) present in compound **1**. The whole structure of compound 1 was then determined, starting from characteristic signals, by means of correlation spectroscopy (COSY), heteronuclear single quantum correlation (HSQC), and heteronuclear multiple bond correlation (HMBC) NMR correlations (Figures S3–S5). The COSY spectrum (Figure 2) showed cross-peaks with signals at H-1 (δ_H_ 2.43, 2.56)/H-10 (δ_H_ 2.00); H-6 (δ_H_ 3.84)/H-7 (δ_H_ 1.61, 1.70)/H-8 (δ_H_ 1.76)/Me-17 (δ_H_ 0.90); H-14 (δ_H_ 7.09)/H-15 (δ_H_ 4.77). Moreover, the key HMBC correlations (Figure 2) of H-1 with C-2, H-3 with C-1, C-4, C-5, and C-18; Me-19 with C-4, C-5, C-6, and C-10, Me-17 with C-7, C-8, and C-9, Me-20 with C-9, C-10, and C-11, and methoxy proton with C-18 led to the construction of the decalin core of compound **1**, including a hydroxy group at C-6 and a methyl ester substituted at C-4. The linkage between C-12 and butanolide via C-13 was established by comparing the corresponding NMR data with those of similar analogues and confirmed by mass spectrometry analysis [22,24,27]. The planar structure of compound **1** is represented in Figure 2. The relative stereochemistry of compound **1** was deduced from nuclear overhauser effect spectroscopy (NOESY) correlations (Figure 2 and Figure S6) and by comparison of its spectroscopic data with those of clerodane analogues. The NOESY experiment showed correlations of H-6 (δ_H_ 3.84)/H-10 (δ_H_ 2.00)/H-8 (δ_H_ 1.76), which indicated protons located on the β face of the molecule. On the other hand, Me-20 presented NOESY correlations with Me-19 and Me-17, but neither Me-19 nor Me-20 correlated with H-10, suggesting that compound **1** is an *ent*-clerodane-type molecule with *trans*-decalin core [28]. The *trans* A/B ring junction was also evidenced by the carbon chemical shifts of C-19 (δ_C_ 14.0) and C-20 (δ_C_ 17.3) [29,30,31]. Thus, these correlations indicated that the hydroxy group at C-6 had an α-configuration, as confirmed by the coupling constants of H-6 with H-7α (*J* = 12.6 Hz) and H-7β (*J* = 4.4 Hz) [32,33]. All the spectral data appeared thus to be in agreement with the structure and stereochemistry of compound **1**.

**Figure 3:**
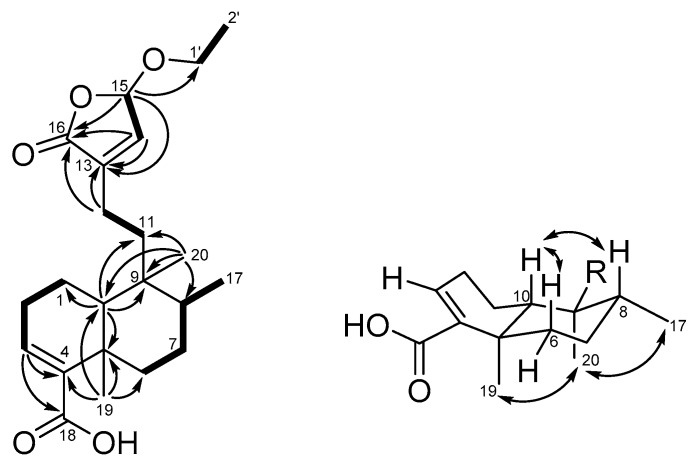
COSY (bold bond), selected HMBC (arrow), and NOESY (left-right arrow) correlations of compound **2**.

Callihypolin B (**2**) was isolated as a yellow oil. It possesses the molecular formula C_22_H_32_O_5_, corresponding to seven indices of hydrogen deficiency, as determined by the HRESIMS ion at *m/z* 399.21419 [M + Na]^+^ (calcd. 399.21420) (Figure S14) and ^13^C NMR data. The IR spectrum revealed the presence of ester (1768 cm^−1^) and conjugated carbonyl (1682 cm^−1^) groups. The ^1^H NMR data of compound **2** (Table 1, Figure S7) demonstrated the presence of one ethoxy [δ_H_ 3.94 (m) and 3.74 (m); 1.27 (t, *J* = 7.1 Hz)], one secondary methyl [δ_H_ 0.81 (d, *J* = 6.2 Hz)], two tertiary methyls (δ_H_ 0.76 and 1.23), and two olefinic methines [δ_H_ 6.85 (m), and 6.76 (d, *J* = 1.2)], together with one hemiacetal methine [δ_H_ 5.79 (brd, *J* = 1.2)]. The ^13^C NMR and DEPT spectra (Table 1, Figure S8) of compound **2** showed the presence of 22 carbon signals ascribable to 4 methyls, 7 methylenes (of which one was oxygenated), 2 olefinic methines, 3 aliphatic methines, 2 aliphatic quaternary carbons, 2 olefinic quaternary carbons, and 2 carbonyl carbons. Two carbonyls and two C=C double bonds accounted for four indices of hydrogen deficiency, so the remaining three indices suggested that compound **2** was a tricyclic compound. In the ^1^H-^1^H COSY spectrum (Figure S9), the correlations of H_2_-1/H_2_-2/H_2_-3, H_2_-6/H_2_-7/H-8/Me-17, H_2_-11/H_2_-12, H-14/H-15, and H_2_-1′/Me-2′ were used to establish the presence of five fragments, as shown in Figure 3. In the HMBC spectrum (Figure 3, Figure S11), the cross-peaks of H-3 with C-4 and C-18; of Me-19 with C-4, C-5, C-6, and C-10; and of H-10 with C-1 and C-5 revealed the presence of a cyclohexene ring (ring A), in which a carboxyl group and Me-19 were attached to C-4 and C-5, respectively. The presence of a cyclohexane ring (ring B) with Me-20 attached at C-9 was elucidated by the HMBC correlations of Me-20 to C-8, C-9, and C-10, as well as of H-10 to C-9. Additionally, both H_3_-20 and H-10 showed correlations with C-11 and indicated the linkage between ring B and C-11 via C-9. The HMBC cross-peaks of H-14 to C-13 (δ_C_ 139.0) and C-16 (δ_C_ 171.5); H-15 (δ_H_ 5.79) to C-16 and C-1′ (δ_C_ 66.0), as well as H_2_-12 to C-13 and C-16, revealed the presence of an α,β-unsaturated γ-lactone ring with an ethoxy group located at C-15. Thus, the planar structure of compound **2** could be established. The stereochemistry of compound **2** was determined by its NOESY spectrum, relative NMR data, and circular dichroism spectrum. The NOESY experiments (Figure 3 and Figure S12) carried out on compound **2** showed correlations of Me-19/Me-20/Me-17, and H-6β (δ_H_ 2.44)/H-10/H-8, whereas no correlation was revealed between H-10 and Me-19. These data, as well as the carbon chemical shift of Me-19 at δ_C_ 20.5 [29], indicated that compound **1** is characterized by a type TC clerodane skeleton under a chair conformation of ring B [34], a *trans* relationship between rings A and B, *α*-orientations of Me-17, Me-19, and Me-20, and β-orientation of H-10. The ethoxy group attached at C-15 in the butenolide moiety was assigned to the α-face by comparison with the circular dichroism (CD) data of known butenolides and by applying the octant rule. The CD spectrum showed a negative Cotton effect near 243 nm (π-π*) and supported the *S* configuration of C-15 [31,35,36]. Thus, the structure and stereochemistry of compound **2** were clearly determined.

**Figure 4:**
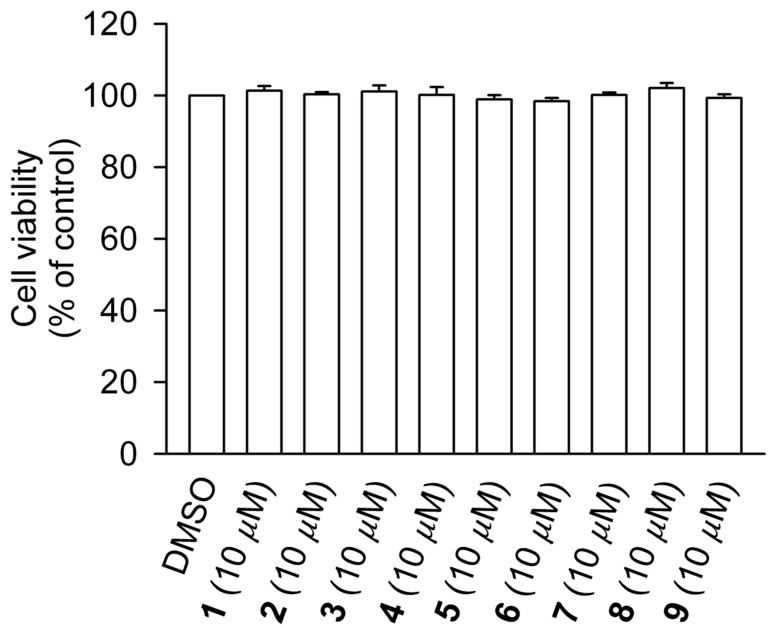
Compounds **1**–**9** do not cause LDH release in human neutrophils. Human neutrophils were incubated with DMSO (as a control) or compounds **1**–**9** (10 μM) for 15 min. Cytotoxicity was evaluated by LDH release. All data are presented as the means ± S.E.M. (*n* = 3).

Compounds **1**–**9** were evaluated for their inhibitory activities on superoxide anion generation and elastase release in formyl-methionyl-leucyl-phenylalanine (fMLF)/cytochalasin (CB)-induced human neutrophils. The formyl peptide fMLF in combination with the priming agent CB serves as a stimulator that mimics the over-activation of neutrophils by a pathogen or an immune system reaction [37]. As shown in Table 2, compounds **2**–**4** exerted anti-inflammatory activity by suppressing superoxide anion generation and elastase release. The positive control genistein, which acts via inhibition of protein tyrosine kinases, showed a profound effect on the respiratory burst (89% inhibition of superoxide generation) and only a mild effect on degranulation (22.8% inhibition of elastase release). Among the tested samples, the new compound **2** showed the best activity, suppressing 32.2% of superoxide generation and 17.6% of elastase release. To exclude possible toxicity to the cells, the lactate dehydrogenase (LDH) release assay was employed, and none of the tested clerodane diterpenoids resulted toxic to human neutrophils (Figure 4). Clerodane diterpenes with an open lactone ring at C16 were previously reported to exert inhibitory effects on the function of neutrophils activated by fMLF/CB, including respiratory burst [38] and degranulation [39]. Thus, our results well correlate with the anti-inflammatory effects of previously isolated clerodane diterpenes and indicate the potential of the new compounds for the development of anti-inflammatory drugs targeting neutrophils.

## 3. Experimental

### 3.1. General

Silica gel 60 (Merck) was used for open-column chromatography (CC). Luna C_18_ (5 m, 250 × 10 mm, Phenomenex), Luna CN (5 m, 250 10 mm, Phenomenex), and Luna phenyl-hexyl (5 m, 250 × 10 mm, Phenomenex) semi-preparative columns were used for high-performance liquid chromatography (HPLC). HPLC used a Shimadzu LC-10AT pump with an SPD-20A UV-Vis detector. The UV spectra were obtained by using a Jasco UV-530 ultraviolet spectrophotometer (Jasco, Tokyo, Japan), whereas the IR spectra were obtained on a Jasco FT-IR-4600 spectrophotometer (Jasco, Tokyo, Japan). Optical rotations were measured with a Jasco P-1020 digital polarimeter (Jasco, Tokyo, Japan). NMR spectra were obtained using JEOL JNM ECS 400 MHz (JEOL, Tokyo, Japan) and Varian 600 MHz NMR spectrometers (Varian, Palo Alto, CA, USA). ESI–MS data were collected on a VG Biotech Quattro 5022 mass spectrometer (VG Biotech, Altrincham, UK). High-resolution ESI–MS data were obtained with a Bruker APEX II spectrometer (Bruker, Bremen, Germany). Circular dichroism spectra were recorded on a JASCO J-810 spectrophotometer (Jasco, Tokyo, Japan).

### 3.2. Plant Material

The plant samples of *C. hypoleucophylla* were collected in Kaohsiung city, Taiwan, in May 2018. The plant material was identified by one of the authors, Dr. Ming-Hong Yen. A voucher sample (specimen code: CH001) was deposited at the Graduate Institute of Natural Products, College of Pharmacy, Kaohsiung Medical University, Kaohsiung, Taiwan.

### 3.3. Extraction and Isolation

Air-dried leaves and twigs of *C. hypoleucophylla* (17.0 kg) were extracted three times with 95% ethanol at room temperature for 72 h each time. The extract was evaporated under reduced pressure to get a crude extract (3.6 kg). Next, the ethanol extract of *C. hypoleucophylla* was suspended and dissolved in H_2_O and then partitioned with ethyl acetate to obtain an ethyl acetate layer (118.3 g). The the ethyl acetate layer was further partitioned between hexanes and 75% MeOH to acquire hexanes and MeOH layers, respectively.

Due to the results of the cytotoxic assay, the MeOH layer (45.6 g) was selected for further isolation. At first, it was loaded on a normal-phase silica gel open column and was eluted by stepwise hexanes with ethyl acetate (1:0~0:1) followed by stepwise ethyl acetate with methanol (1:0~0:1) to obtain seven subfractions (CH1~7), according to TLC analysis. The third sub-fraction, CH3, was isolated on Sephadex LH-20 and eluted with MeOH to afford four subfractions (CH3-1–4). Then, repeated column chromatography isolation on CH3-3 yielded CH3-3-1–5 fractions. CH3-3-2 (500.3 mg) was separated by silica gel CC (dichloromethane/MeOH, 100:1→0:1) to afford more subfractions (CH3-3-2-1–6). Fr. CH3-3-2-6 was purified by normal-phase HPLC using a Phenomenex Luna-CN column (hexane/dichloromethane/methanol, 30:10:1, 1.5 mL/min) to give compounds **2** (33.1mg), **3** (7.2 mg), **4** (62.7 mg), and 5 (26.7 mg). Fr. CH3-3-2-4 was isolated by reverse-phase HPLC using a CN column and gave compounds **1** (1.9 mg) and 7 (0.7 mg). Fr. CH3-3-3 was subjected to silica gel CC (CH_2_Cl_2_/MeOH, 1:0→0:1) followed by NP-CN HPLC and elution with (hexane/dichloromethane/methanol, 40:10:1, 2.0 mL/min) to obtain compound **8** (9.8 mg). In addition, Fr. CH3-2 was separated by normal-phase silica gel CC with hexane/dichloromethane/methanol (100:40:1→0:0:1) to afford Frs. CH3-2-1–5. Fr. CH3-2-5 was purified by silica gel CC (CH_2_Cl_2_/MeOH, 1:0→0:1) followed by RP-phenyl-hexyl HPLC (methanol/H_2_O, 65/35, 2.0 mL/min) to give compounds **6** (2.5 mg) and **9** (7.0 mg).

### 3.4. Spectroscopic Data

Callihypolin A (**1**) yellow oily, ${\left[\alpha \right]}_{D}^{26}$ −1.0° (*c* 0.05, MeOH); IR (neat) ν_max_ 3452, 2956, 1768, 1682, 1376, 1342, 1202, 1141, 1018 cm^−1^; ^1^H-NMR and ^13^C-NMR (CDCl_3_, 600/150 MHz) see Table 1; HRESIMS *m/z* 399.17785 (calcd for C_21_H_28_O_6_Na, 399.17781).

Callihypolin B (**2**) yellow oily, ${\left[\alpha \right]}_{D}^{26}$ −47.6° (*c* 0.05, MeOH); IR (neat) *ν*_max_ 3451, 2930, 1739, 1678, 1450, 1253, 1072 cm^−1^; ^1^H-NMR and ^13^C-NMR (CDCl_3_, 400/100 MHz) see Table 1; HRESIMS *m/z* 399.21419 (calcd for C_22_H_32_O_5_Na, 399.21420).

### 3.5. Superoxide Anion Generation and Elastase Release Assays by Human Neutrophils

Human neutrophils were obtained from the venous blood of healthy adult volunteers (20−30 years old), following a reported procedure [37]. Superoxide anion generation by fMLF (0.1 μM)/CB (1μM)-activated neutrophils was evaluated based on the reduction of ferricytochrome c, as previously described [37,40]. Elastase release by the fMLF (0.1 μM)/CB (0.5 μM)-activated neutrophils was determined using N-methoxysuccinyl-Ala-Ala-Pro-Val-p-nitroanilide as the elastase substrate, according to a previous protocol [37,40]. The concentration was 10 μM for compounds **1**–**9**. Genistein was used as a positive control.

### 3.6. Cytotoxicity Test

A lactate dehydrogenase (LDH) assay kit (Promega, Madison, WI, USA) was utilized to evaluate the cytotoxicity of the samples in human neutrophils. Human neutrophils were treated with DMSO or compounds **1**–**9** for 15 min at 37 °C. Cell-free supernatants were collected, and the amount of LDH was evaluated [37].

## 4. Conclusions

The first phytochemical investigation of the leaves and twigs of the Taiwanese endemic plant *Callicarpa hypoleucophylla* has resulted in the isolation of nine clerodane-type diterpenoids, compounds **1**–**9**, including two new compounds designated callihypolins A and B (compounds **1** and **2**). All isolates from *C. hypoleucophylla* possess a TC *ent*-clerodane skeleton, which is different from that of the phyllocladane and labdane diterpenoids that were identified as major components of the other well-studied species *Callicarpa macrophylla* Vahl, which is recorded in the Pharmacopoeia of the People’s Republic of China. These results reflect the unique properties of *C. hypoleucophylla* from the perspective of chemotaxonomy. Moreover, the anti-inflammatory activity of the isolated compounds highlights the potential of clerodane-type diterpenoids for further pharmaceutic development.

## Figures and Tables

**Table 1:** ^1^H and ^13^C NMR Data of compounds **1** and **2** in CDCl_3_.

	1 *^a^*		2 *^b^*	
Position	δ_H_ Mult. (*J* in Hz)	δ_C_, Type	δ_H_ Mult. (*J* in Hz)	δ_C_, Type
1	2.56 (dd, 18.0, 14.2)	34.4, CH_2_	1.46 (m)	17.3, CH_2_
	2.43 (dd, 18.0, 3.5)		1.66 (m)	
2		199.0, C	2.27 (m)	27.4, CH_2_
3	5.96 (s)	126.6, CH	6.85 (m)	140.3, CH
4		160.4, C		141.2, C
5		45.5, C		37.5, C
6	3.84 (dd, 12.6, 4.4)	72.5, CH	2.44 (m)	35.7, CH_2_
			1.14 (m)	
7	1.70 (dt, 12.6, 4.4)	36.1, CH_2_	1.46 (m)	27.2, CH_2_
	1.61 (m)		1.42 (m)	
8	1.76 (m)	34.5, CH	1.50 (m)	36.2, CH
9		38.5, C		38.7, C
10	2.00 (dd, 14.2, 3.5)	44.9, CH	1.32 (d, 11.6)	46.6, CH
11	1.61 (m)	34.8, CH_2_	1.50 (m)	35.7, CH_2_
	1.47 (m)		1.66 (m)	
12	2.19 (m)	18.7, CH_2_	2.20 (m)	18.9, CH_2_
	2.00 (tm, 13.0)		2.03 (m)	
13		134.0, C		139.0, C
14	7.09 (quin, 1.7)	143.9, CH	6.76 (quin, 1.2)	141.4, CH
15	4.77 (dd, 3.9, 1.7)	70.2, CH_2_	5.79 (brd, 1.2)	101.6, CH
16		174.0, C		171.5, C
17	0.90 (d, 6.8)	15.3, CH_3_	0.81 (d, 6.2)	15.9, CH_3_
18		169.8, C		172.3, C
19	1.33 (s)	14.0, CH_3_	1.23 (s)	20.5, CH_3_
20	0.84 (s)	17.3, CH_3_	0.76 (s)	18.2, CH_3_
1′	3.81 (s)	52.8, CH_3_	3.94 (m)	66.0, CH_2_
			3.74 (m)	
2′			1.27 (t, 7.1)	15.0, CH_3_

*^a^*^1^H and ^13^C-NMR were measured at 600 and 150 MHz. *^b^*^1^H and ^13^C-NMR were measured at 400 and 100 MHz.

**Table 2:** Inhibitory effects of compounds **1**–**9** on superoxide anion generation and elastase release in formyl-methionyl-leucyl-phenylalanine (fMLF)/ cytochalasin (CB)-induced human neutrophils.

Compound	Superoxide Anion		Elastase Release	
	Inh %		Inh %	
**1**	20.28 ± 5.98	*	8.26 ± 3.72	
**2**	32.19 ± 6.92	**	17.55 ± 2.64	***
**3**	31.19 ± 5.99	**	12.15 ± 2.38	***
**4**	32.88 ± 4.41	***	13.57 ± 1.48	***
**5**	23.65 ± 7.67	*	7.33 ± 1.56	**
**6**	8.44 ± 6.40		10.50 ± 3.23	*
**7**	7.93 ± 5.86		9.30 ± 2.91	*
**8**	15.23 ± 6.37		11.80 ± 3.55	*
**9**	18.80 ± 7.82		16.30 ± 3.74	**
**Genistein ^a^**	89.00 ± 3.00	***	22.79 ± 2.25	***

Percentage of inhibition (Inh %) at 10 μM concentration. Results are presented as mean ± S.E.M. (n = 4–5); * *p* < 0.05, ** *p* < 0.01, *** *p* < 0.001 compared with the control (solvent). ^a^ Genistein served as a positive control.

## Supplementary Materials

The NMR spectra of compounds **1** and **2** are available online.

## References

[1] Tu Y., Sun L., Guo M., Chen W. (2013). The medicinal uses of *Callicarpa,* L. in traditional Chinese medicine: An ethnopharmacological, phytochemical and pharmacological review. J. Ethnopharmacol..

[2] Kawazu K., Inaba M., Mitsui T. (1967). Studies on fish-killing components of *Callicarpa candicans*. Agr. Biol. Chem..

[3] Cantrell C.L., Klun J.A., Bryson C.T., Kobaisy M., Duke S.O. (2005). Isolation and identification of mosquito bite deterrent terpenoids from leaves of American (*Callicarpa americana*) and Japanese (*Callicarpa japonica*) beautyberry. J. Agric. Food Chem..

[4] Zhang L., Dong L., Huang J., Liu M., Li G., Zhang C., Zhang K., Wang J. (2013). 3,4-*seco*-Labdane diterpenoids from the leaves of *Callicarpa nudiflora* and their inhibitory effects on nitric oxide production. Fitoterapia.

[5] Dong L., Zhang L., Zhang X., Liu M., Wang J., Wang Y. (2014). Two new 3,4-*seco*-labdane diterpenoids from *Callicarpa nudiflora* and their inhibitory activities against nitric oxide production. Phytochem. Lett..

[6] Cheng H.H., Cheng Y.B., Hwang T.L., Kuo Y.H., Chen C.H., Shen Y.C. (2015). Randainins A–D, based on unique diterpenoid architectures, from *Callicarpa randaiensis*. J. Nat. Prod..

[7] Zhou Z., Wei X., Fu H., Luo Y. (2013). Chemical constituents of *Callicarpa nudiflora* and their anti-platelet aggregation activity. Fitoterapia.

[8] Wu A.Z., Zhai Y.J., Zhao Z.X., Zhang C.X., Lin C.Z., Zhu C.C. (2013). Phenylethanoid glycosides from the stems of *Callicarpa peii* (hemostatic drug). Fitoterapia.

[9] Luo Y.H., Zhou Z.Q., Ma S.C., Fu H.Z. (2014). Three new antioxidant furofuran lignans from *Callicarpa nudiflora*. Phytochem. Lett..

[10] Cai H., Xie Z., Liu G., Sun X., Peng G., Lin B., Liao Q. (2014). Isolation, identification and activities of natural antioxidants from *Callicarpa kwangtungensis* Chun. PLoS ONE.

[11] Jones W.P., Lobo-Echeverri T., Mi Q., Chai H.B., Soejarto D.D., Cordell G.A., Swanson S.M., Kinghorn A.D. (2007). Cytotoxic constituents from the fruiting branches of *Callicarpa Americana* collected in southern Florida. J. Nat. Prod..

[12] Mei W.L., Han Z., Cui H.B., Zhao Y.X., Deng Y.Y., Dai H.F. (2010). A new cytotoxic iridoid from *Callicarpa nudiflora*. Nat. Prod. Res..

[13] Xu J., Sun Y., Wang M., Ren Q., Li S., Wang H., Sun X., Jin D.Q., Sun H., Ohizumi Y. (2015). Bioactive diterpenoids from the leaves of *Callicarpa macrophylla*. J. Nat. Prod..

[14] Chen J.J., Wu H.M., Peng C.F., Chen I.S., Chu S.D. (2009). *seco*-Abietane diterpenoids, a phenylethanoid derivative, and antitubercular constituents from *Callicarpa pilosissima*. J. Nat. Prod..

[15] Huang B., Fu H.Z., Chen W.K., Luo Y.H., Ma S.C. (2014). Hepatoprotective triterpenoid saponins from *Callicarpa nudiflora*. Chem. Pharm. Bull..

[16] Luo Y.H., Fu H.Z., Huang B., Chen W.K., Ma S.C. (2016). Hepatoprotective iridoid glucosides from *Callicarpa nudiflora*. J. Asian. Nat. Prod. Res..

[17] Chung P.Y., Chung L.Y., Navaratnam P. (2014). Potential targets by pentacyclic triterpenoids from *Callicarpa farinosa* against methicillin-resistant and sensitive. *Staphylococcus aureus*. Fitoterapia.

[18] Gupta S.K., Gupta A., Gupta A.K., Pakash D.V. (2013). *In vitro* anti-arthritic activity of ethanolic extract of *Callicarpa Macrophylla* flower. Int. Res. J. Pharm..

[19] Yadav V., Jayalakshmi S., Singla R.K., Patra A., Khan S. (2012). Assessment of anti-inflammatory and analgesic activities of *Callicarpa macrophylla* Vahl. roots extracts. Webmed Cent. Pharmacol..

[20] Ahmad V.U., Farooq U., Abbaskhan A., Hussain J., Abbasi M.A., Nawaz S.A., Choudhary M.I. (2004). Four new diterpenoids from *Ballota limbata*. Helv. Chim. Acta..

[21] Pinto M.E.F., Silva M.S.D., Schindler E., Filho J.M.B., El-Bachá R.D.S., Castello-Branco M.V.S., Agra M.D.F., Tavares J.F. (2010). 3′,8"-Biisokaempferide, a cytotoxic biflavonoid and other chemical constituents of *Nanuza plicata* (Velloziaceae). J. Braz. Chem. Soc..

[22] Farooq U., Khan A., Ahmad V.U., Kousar F., Iqbal S. (2005). Limbatolide F and G: Two new *trans*-clerodane diterpenoids from *Otostegia limbata*. Pol. J. Chem..

[23] Ahmad V.U., Khan A., Farooq U., Kousar F., Khan S.S., Nawaz S.A., Abbasi M.A., Choudhary M.I. (2005). Three new cholinesterase-inhibiting *cis*-clerodane diterpenoids from *Otostegia limbata*. Chem. Pharm. Bull..

[24] Iqbal Choudhary M., Mohammad M.Y., Musharraf S.G., Onajobi I., Mohammad A., Anis I., Shah M.R., Atta-Ur-Rahman (2013). Biotransformation of clerodane diterpenoids by *Rhizopus stolonifer* and antibacterial activity of resulting metabolites. Phytochemistry.

[25] Raha P., Das A.K., Adityachaudhuri N., Majumder P.L. (1991). Cleroinermin, *aneo*-clerodane diterpenoid from *Clerodendron inermi*. Phytochemistry.

[26] Huang Z., Jiang M.Y., Zhou Z.Y., Xu D. (2010). Two new clerodane diterpenes from *Dodonaea viscosa*. Z. Naturforsch..

[27] Song B., Ding G., Tian X.H., Li L., Zhou C., Zhang Q.B., Wang M.H., Zhang T., Zou Z.M. (2015). Anti-HIV-1 integrase diterpenoids from *Dichrocephala benthamii*. Phytochem. Lett..

[28] Rustaiyan A., Simozar E., Ahmadi A., Grenz M., Bohlmann F. (1981). A hardwickiic acid derivative from *Pulicaria gnaphalodes*. Phytochemistry.

[29] Heymann H., Tezuka Y., Kikuchi T., Supriyatna S. (1994). Constituents of *Sindora sumatrana* M_IQ_. III. new *trans*-clerodane diterpenoids from the dried pods. Chem. Pharm. Bull..

[30] García A., Ramírez-Apan T., Cogordan J.A., Delgado G. (2006). Absolute configuration assignments by experimental and theoretical approaches of *ent*-labdane- and *cis*-*ent*-clerodane-type diterpenes isolated from *Croton glabellus*. Can. J. Chem..

[31] Chang F.R., Huang S.T., Liaw C.C., Yen M.H., Hwang T.L., Chen C.Y., Hou M.F., Yuan S.S., Cheng Y.B., Wu Y.C. (2016). Diterpenes from *Grangea maderaspatana*. Phytochemistry.

[32] Calderón C., De Ford C., Castro V., Merfort I., Murillo R. (2014). Cytotoxic clerodane diterpenes from *Zuelania guidonia*. J. Nat. Prod..

[33] Oberlies N.H., Burgess J.P., Navarro H.A., Pinos R.E., Fairchild C.R., Peterson R.W., Soejarto D.D., Farnsworth N.R., Kinghorn A.D., Wani M.C. (2002). Novel bioactive clerodane diterpenoids from the leaves and twigs of *Casearia sylvestris*. J. Nat. Prod..

[34] Tokoroyama T. (2000). Synthesis of clerodane diterpenoids and related compounds-stereoselective construction of the decalin skeleton with multiple contiguous stereogenic centers. Synthesis.

[35] Wu T.H., Cheng Y.Y., Liou J.R., Way T.D., Chen C.J., Chen Y.H., Kuo S.C., El-Shazly M., Chang F.R., Wu Y.C. (2014). Clerodane diterpenes from *Polyalthia longifolia* var. *pendula* protect SK-N-MC human neuroblastoma cells from β-amyloid insult. RSC Adv..

[36] Itokawa H., Morita H., Katou I., Takeya K., Cavalheiro A.J., de Oliveira R.C., Ishige M., Motidome M. (1988). Cytotoxic diterpenes from the rhizomes of *Hedychium coronarium*. Planta Med..

[37] Liu F.C., Yu H.P., Chen P.J., Yang H.W., Chang S.H., Tzeng C.C., Cheng W.J., Chen Y.R., Chen Y.L., Hwang T.L. (2019). A novel NOX2 inhibitor attenuates human neutrophil oxidative stress and ameliorates inflammatory arthritis in mice. Redox Biol..

[38] Chang F.R., Hwang T.L., Yang Y.L., Li C.E., Wu C.C., Issa H.H., Hsieh W.B., Wu Y.C. (2006). Anti-inflammatory and cytotoxic diterpenes from formosan *Polyalthia longifolia* var. pendula. Planta Med..

[39] Chang H.L., Chang F.R., Chen J.S., Wang H.P., Wu Y.H., Wang C.C., Wu Y.C., Hwang T.L. (2008). Inhibitory effects of 16-hydroxycleroda-3,13(14)*E*-dien-15-oic acid on superoxide anion and elastase release in human neutrophils through multiple mechanisms. Eur. J. Pharmacol..

[40] Yang S.C., Chung P.J., Ho C.M., Kuo C.Y., Hung M.F., Huang Y.T., Chang W.Y., Chang Y.W., Chan K.H., Hwang T.L. (2013). Propofol inhibits superoxide production, elastase release, and chemotaxis in formyl peptide-activated human neutrophils by blocking formyl peptide receptor 1. J. Immunol..

