# Supplementary material for: Clerodane Diterpenoids from Callicarpa hypoleucophylla and Their Anti-Inflammatory Activity
Source: Molecules. 2020 May 13;25(10):2288. doi: 10.3390/molecules25102288 (PMC7287842; doi:10.3390/molecules25102288)
Supplement: Supplementary file 1 [file molecules-25-02288-s001.pdf]

Supporting Information

Clerodane Diterpenoids from *Callicarpa hypoleucophylla* and Their Anti-Inflammatory Activity

Yu-Chi Lin, Jue-Jun Lin, Shu-Rong Chen, Tsong-Long Hwang, Shu-Yen Fang, Michal Korinek, Ching-Yeu Chen, Yun-Sheng Lin, Tung-Ying Wu, Ming-Hong Yen, Chih-Hsin Wang*, and Yuan-Bin Cheng*

Table of Contents

Figure S1 ^{1}H NMR spectrum of callihypolin A (**1**) ($CDCl_3$, 600 MHz) 2
Figure S2 ^{13}C NMR and DEPT spectrum of callihypolin A (**1**) ($CDCl_3$, 150 MHz). 3
Figure S3 COSY spectrum of callihypolin A (**1**) 4
Figure S4 HSQC spectrum of callihypolin A (**1**) 5
Figure S5 HMBC spectrum of callihypolin A (**1**) 6
Figure S6 NOESY spectrum of callihypolin A (**1**) 7
Figure S7 ^{1}H NMR spectrum of callihypolin B (**2**) ($CDCl_3$,400 MHz) 8
Figure S8 ^{13}C NMR and DEPT spectrum of callihypolin B (**2**) ($CDCl_3$, 100 MHz) 9
Figure S9 COSY spectrum of callihypolin B (**2**) 10
Figure S10 HSQC spectrum of callihypolin B (**2**) 11
Figure S11 HMBC spectrum of callihypolin B (**2**) 12
Figure S12 NOESY spectrum of callihypolin B (**2**) 13
Figure S13 HRESIMS spectrum of callihypolin A (**1**) 14
Figure S14 HRESIMS spectrum of callihypolin B (**2**) 15
Figure S15 Representative traces of superoxide anion generation for compounds **2**–**4** 16
Figure S16 Representative traces of elastase release for compounds **2**–**4** 17

Figure S1 ^{1}H NMR spectrum of callihypolin A (**1**) ($CDCl_3$, 600 MHz)

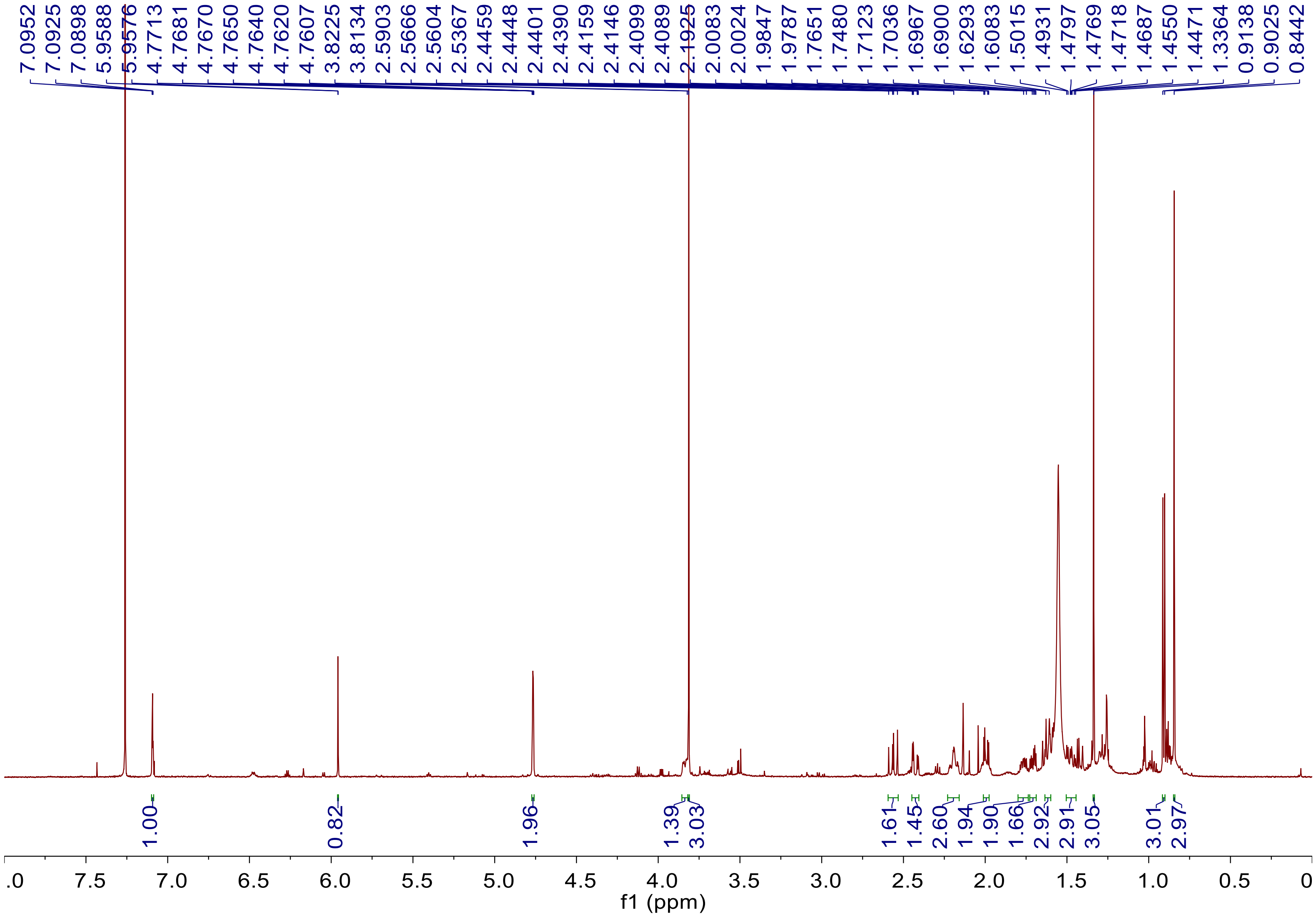

Figure S2 ^{13}C NMR and DEPT spectrum of callihypolin A (**1**) ($CDCl_3$, 150 MHz).

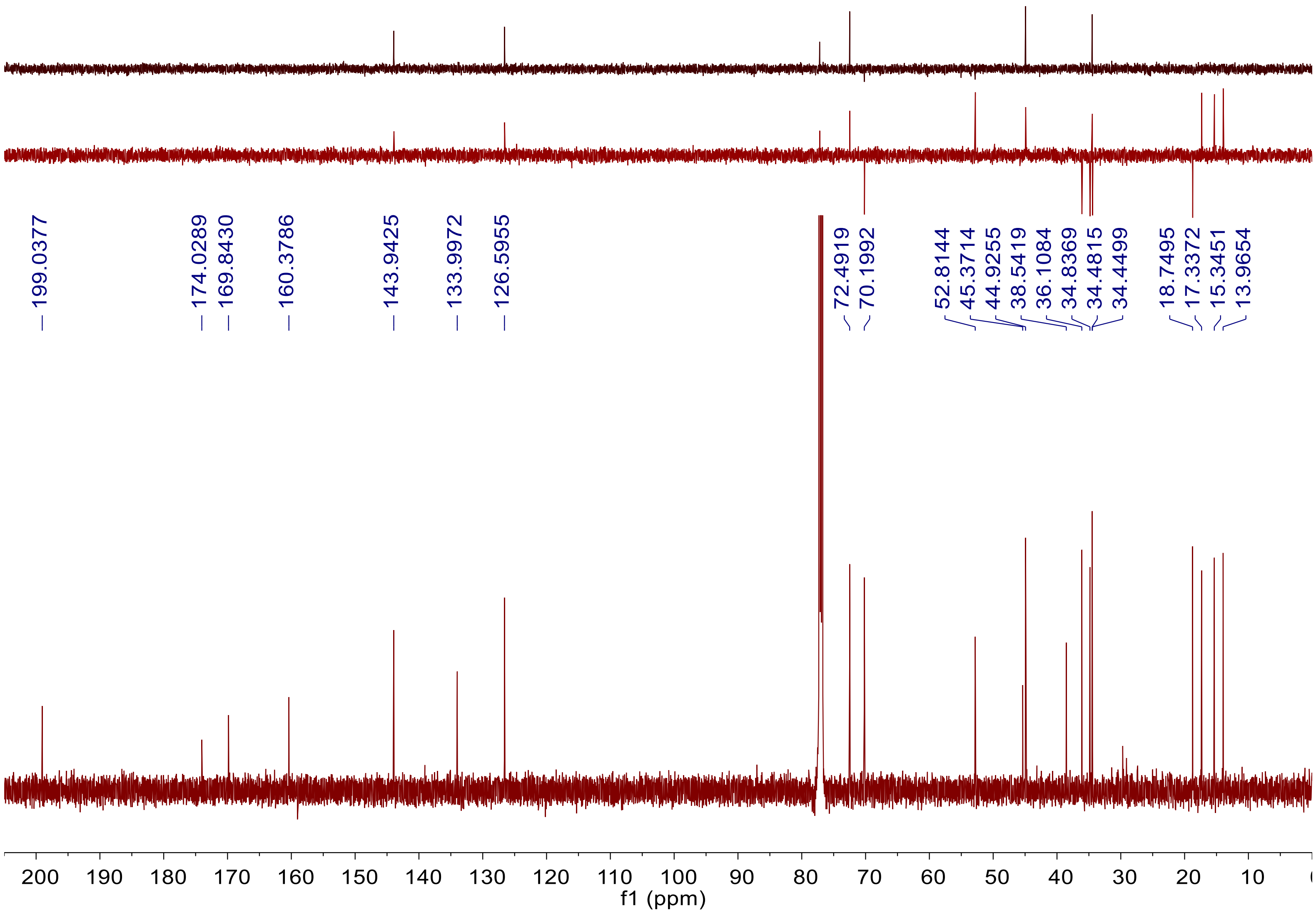

Figure S3 COSY spectrum of callihypolin A (**1**)

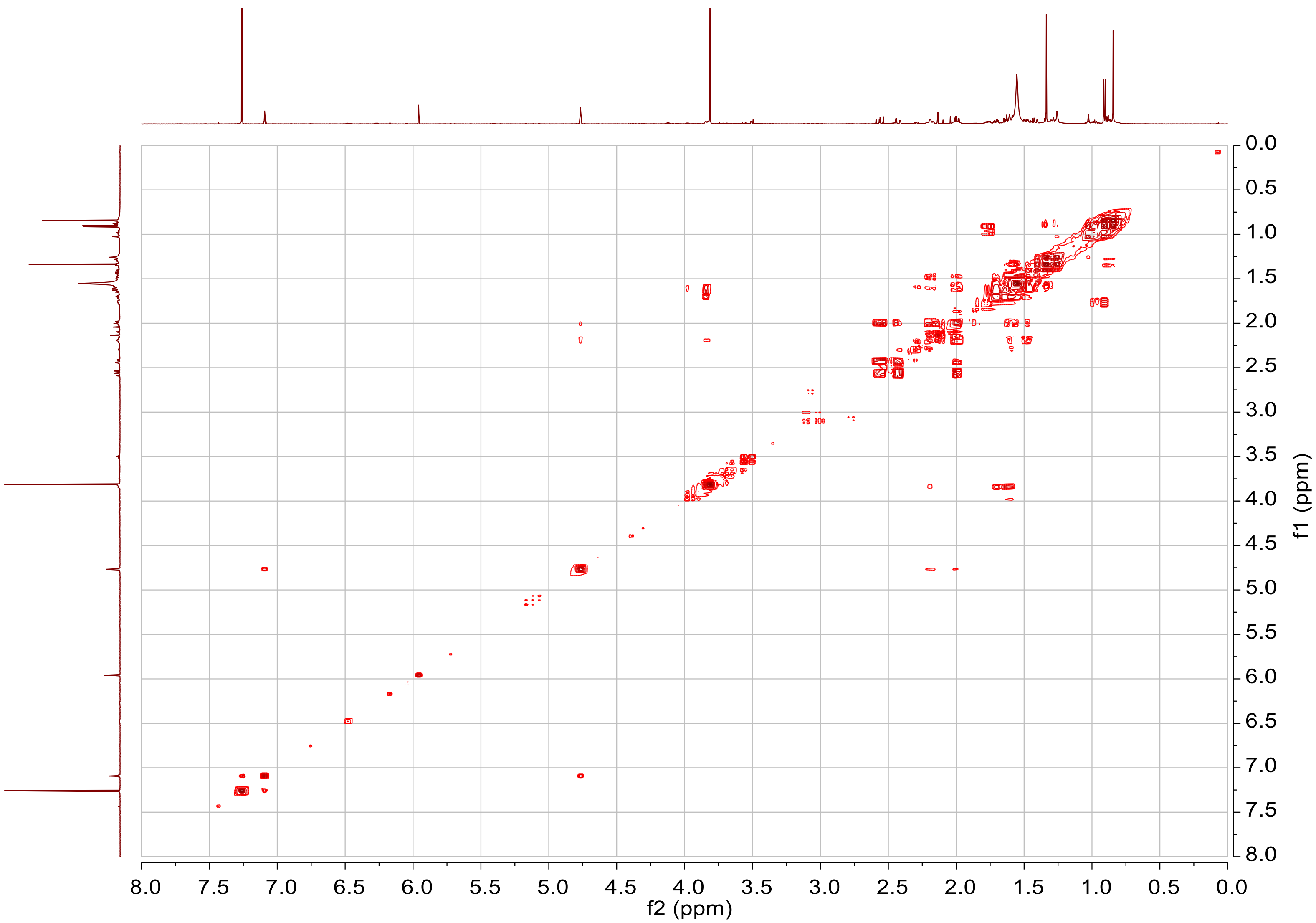

Figure S4 HSQC spectrum of callihypolin A (**1**)

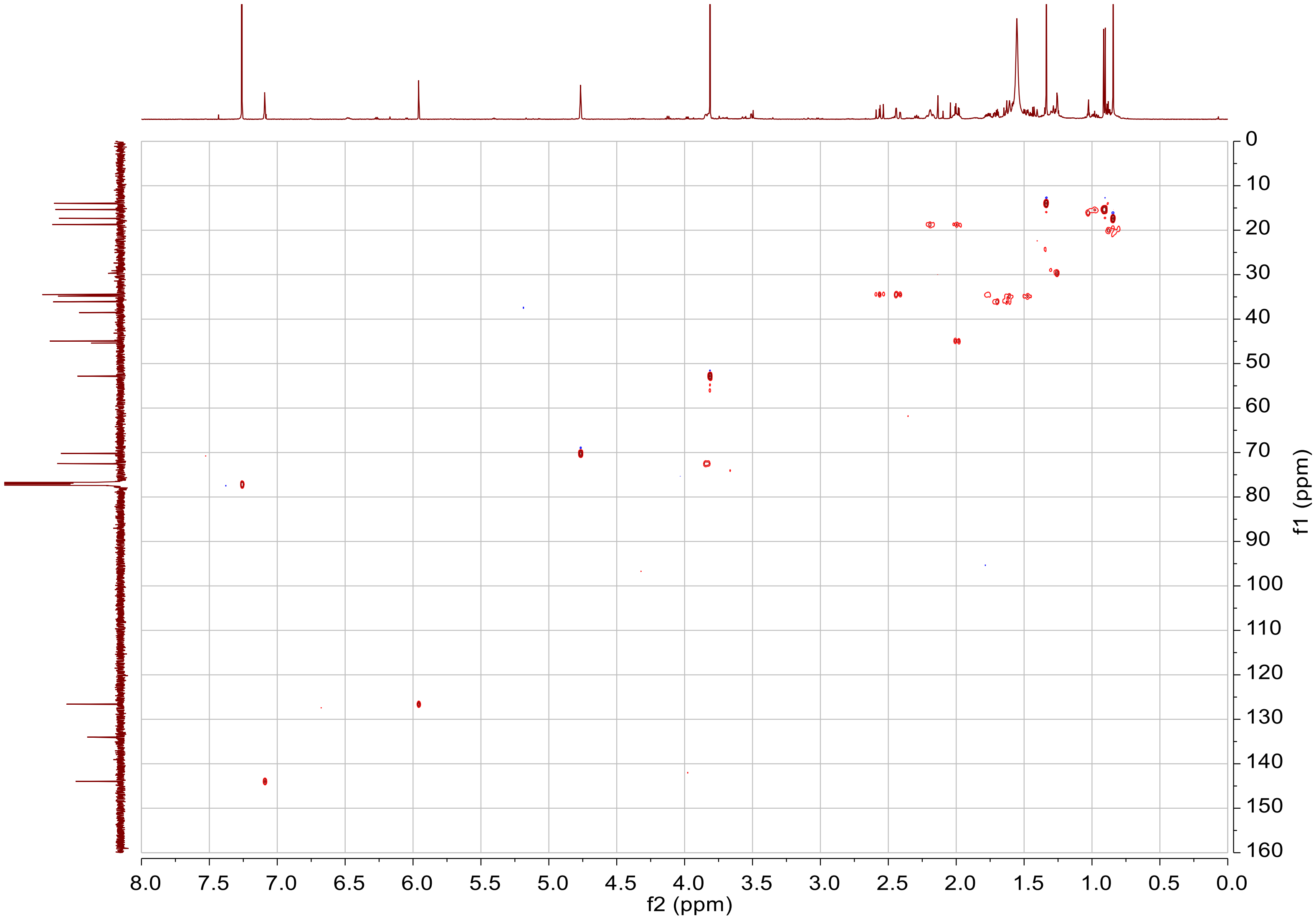

Figure S5 HMBC spectrum of callihypolin A (**1**)

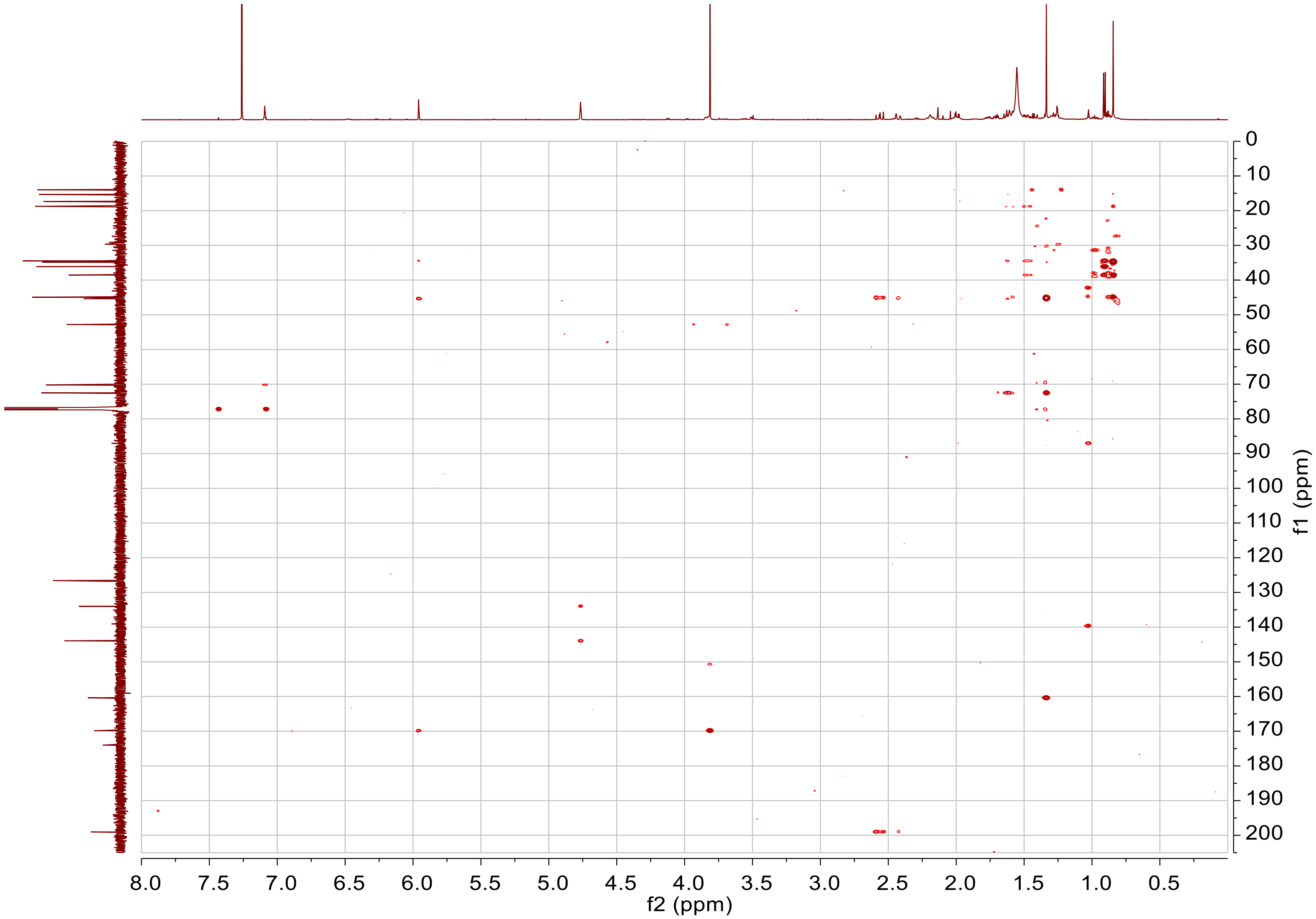

Figure S6 NOESY spectrum of callihypolin A (**1**)

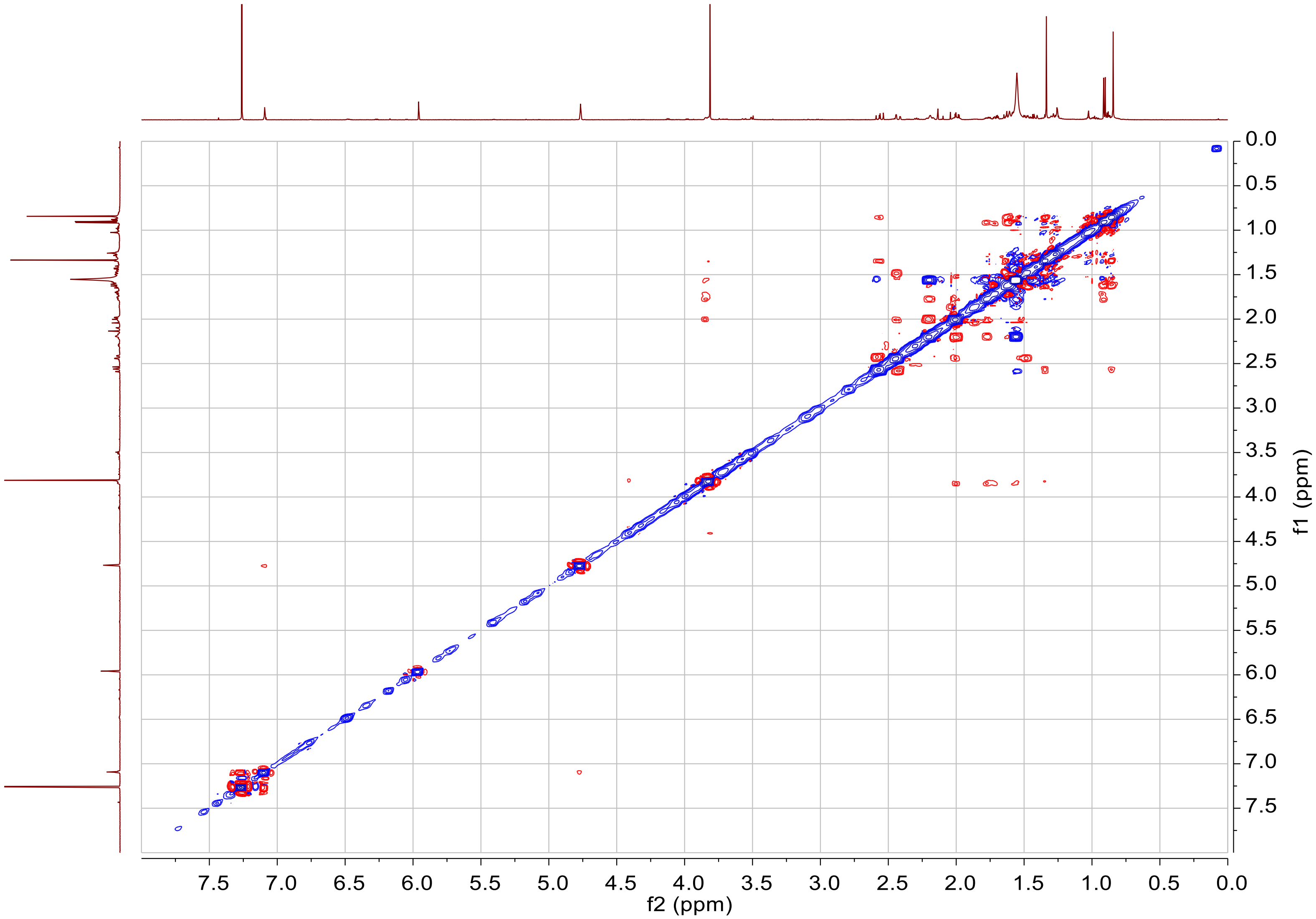

Figure S7 ^{1}H NMR spectrum of callihypolin B (**2**) (CDCl$_3$,400 MHz)

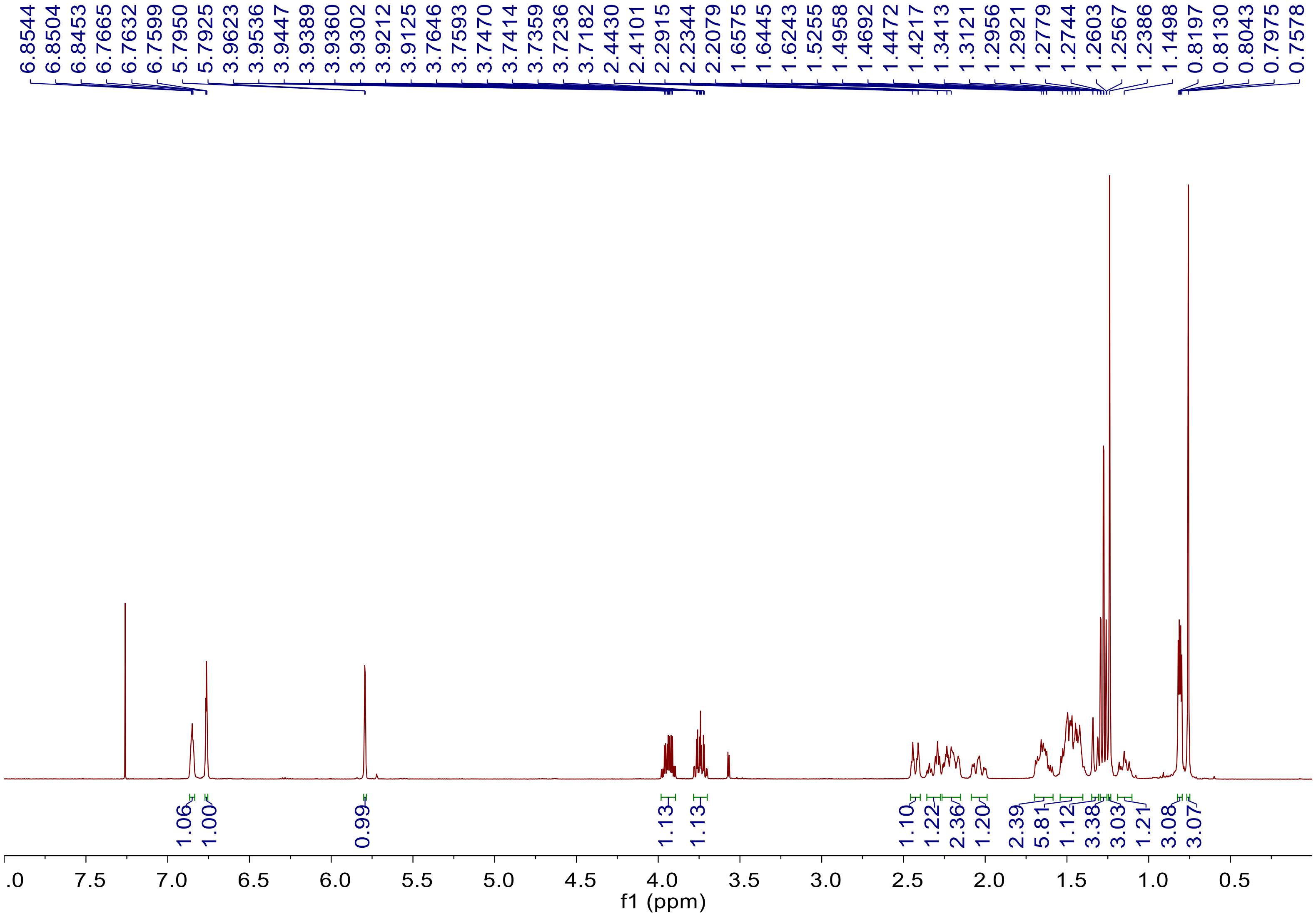

Figure S8 ^{13}C NMR and DEPT spectrum of callihypolin B (**2**) ($CDCl_3$, 100 MHz)

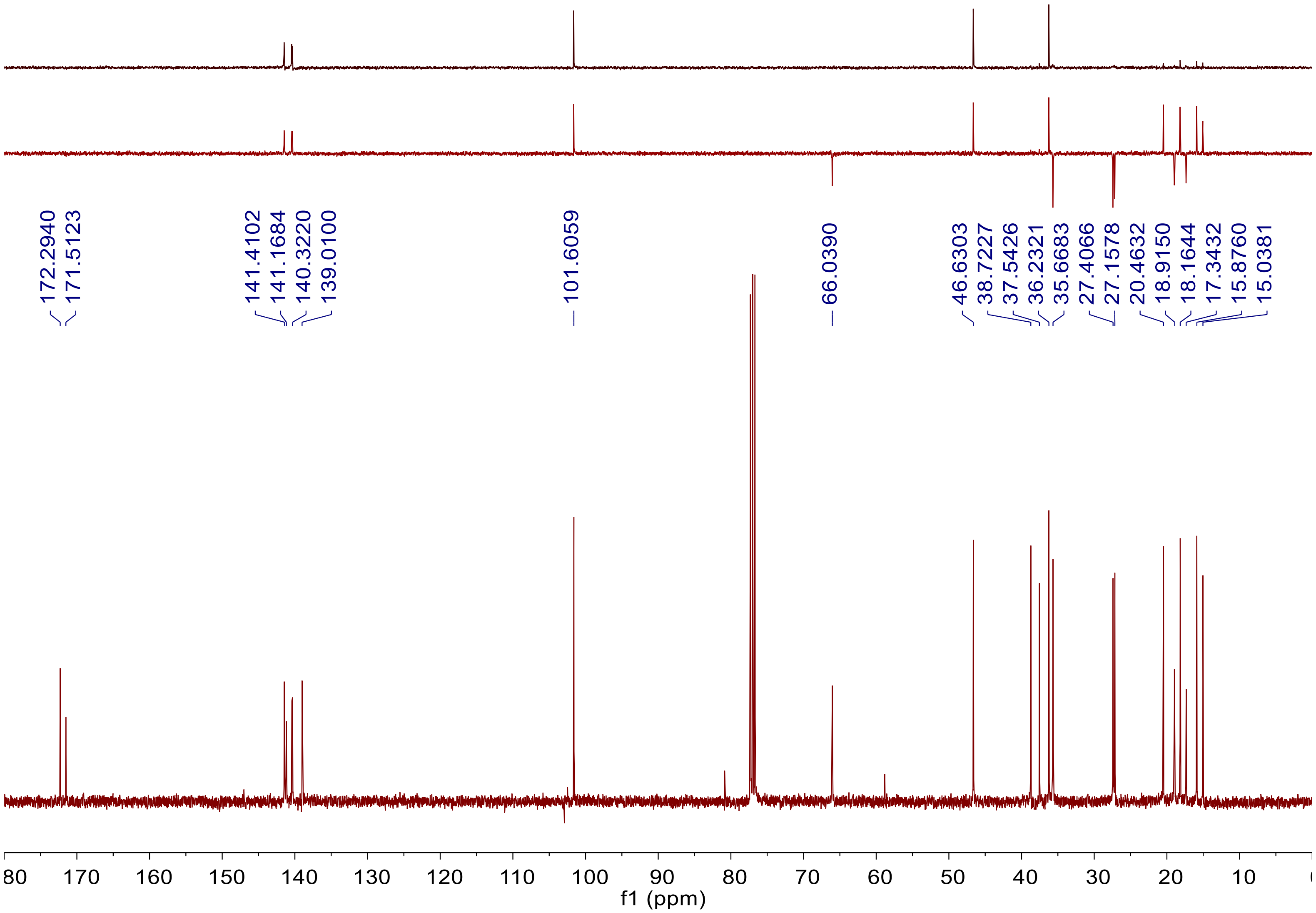

Figure S9 COSY spectrum of callihypolin B (**2**)

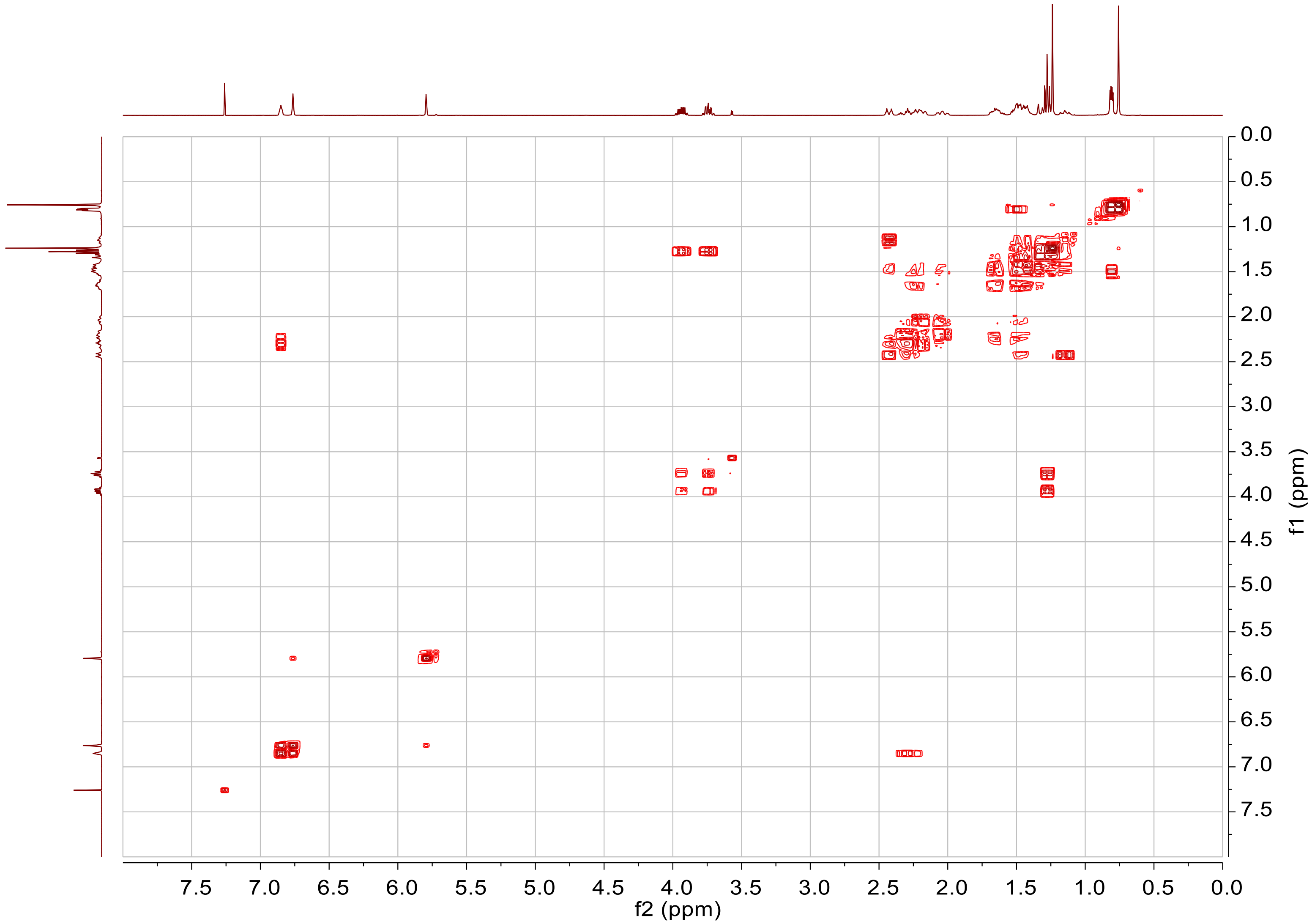

Figure S10 HSQC spectrum of callihypolin B (**2**)

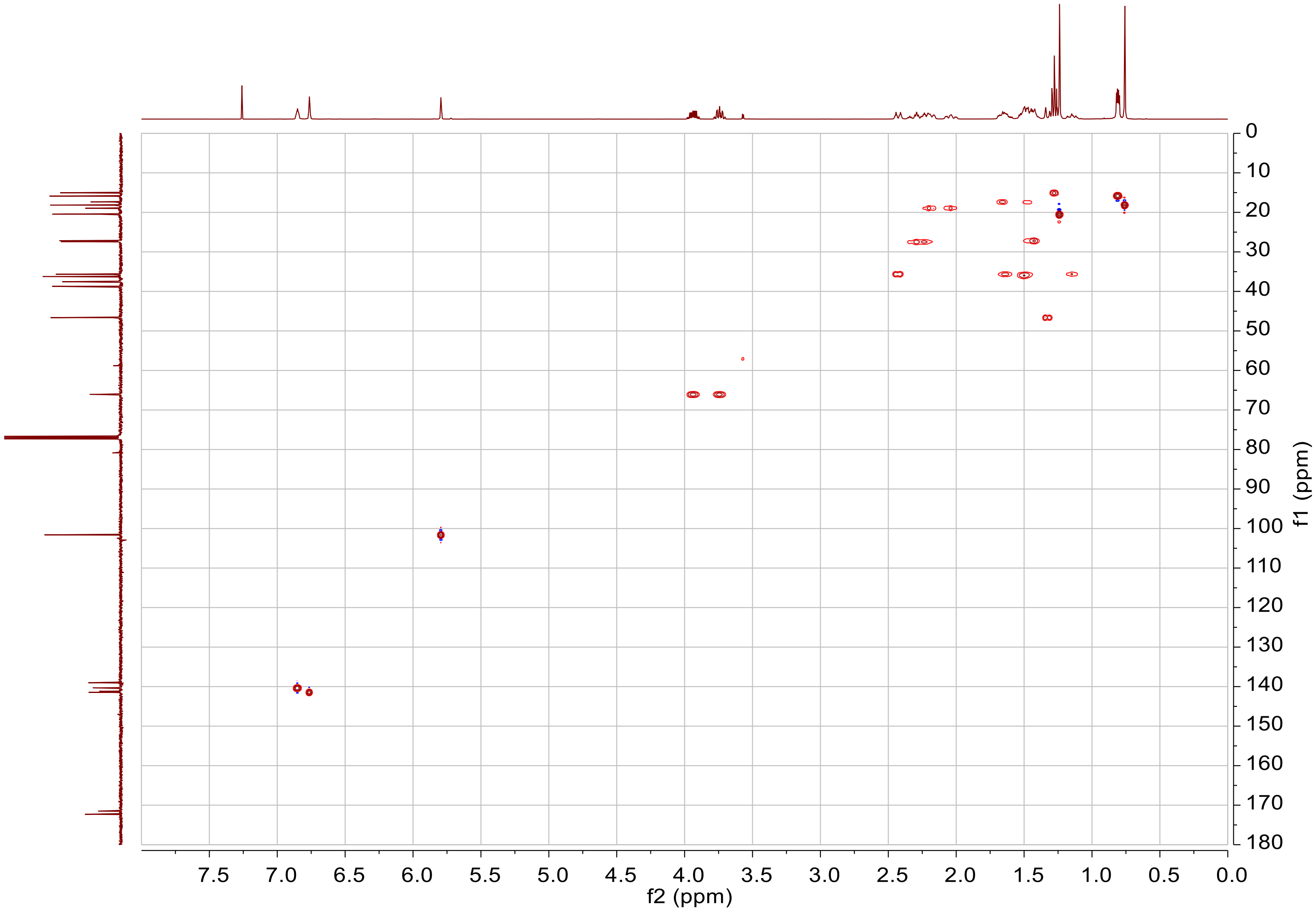

Figure S11 HMBC spectrum of callihypolin B (**2**)

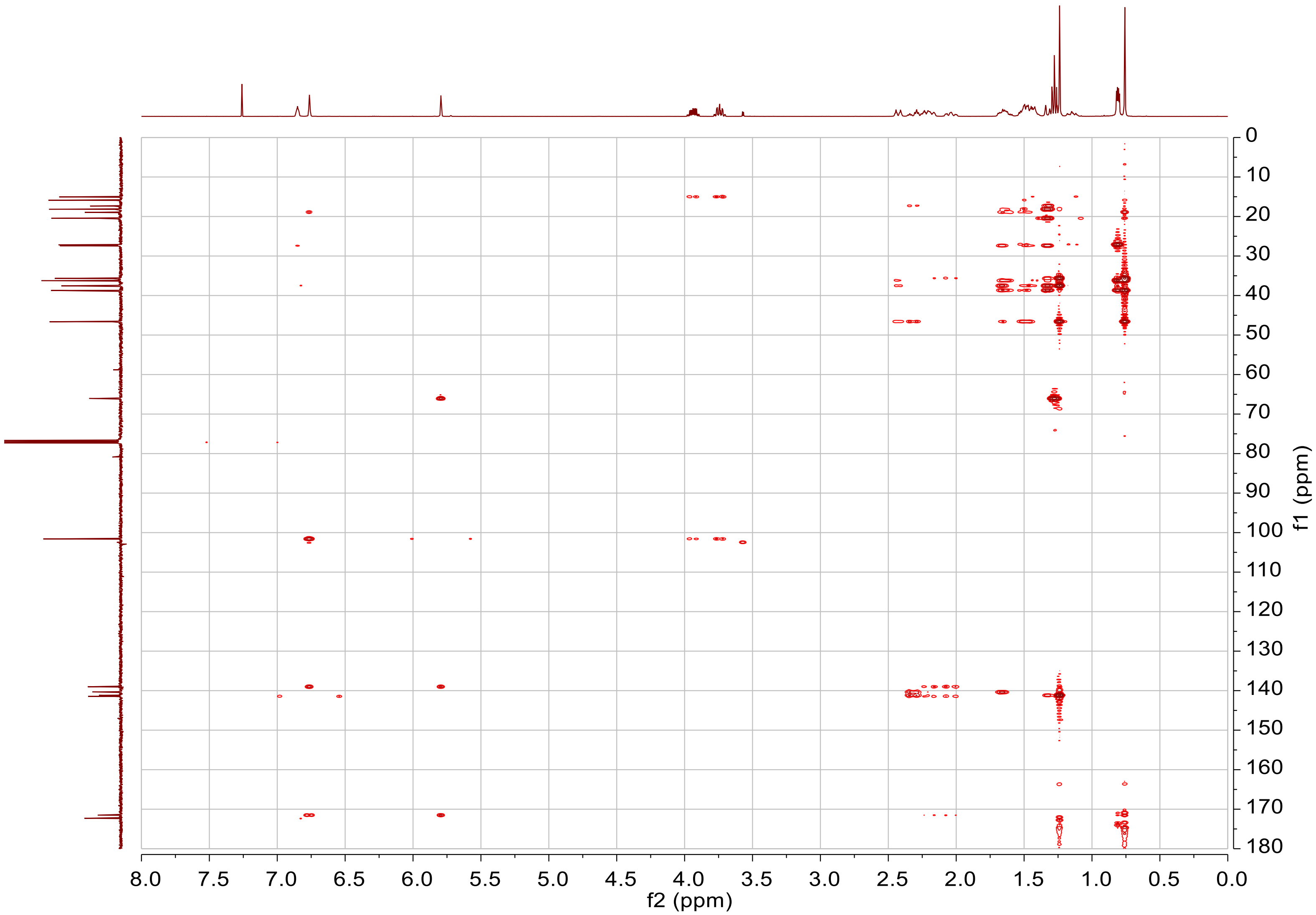

Figure S12 NOESY spectrum of callihypolin B (**2**)

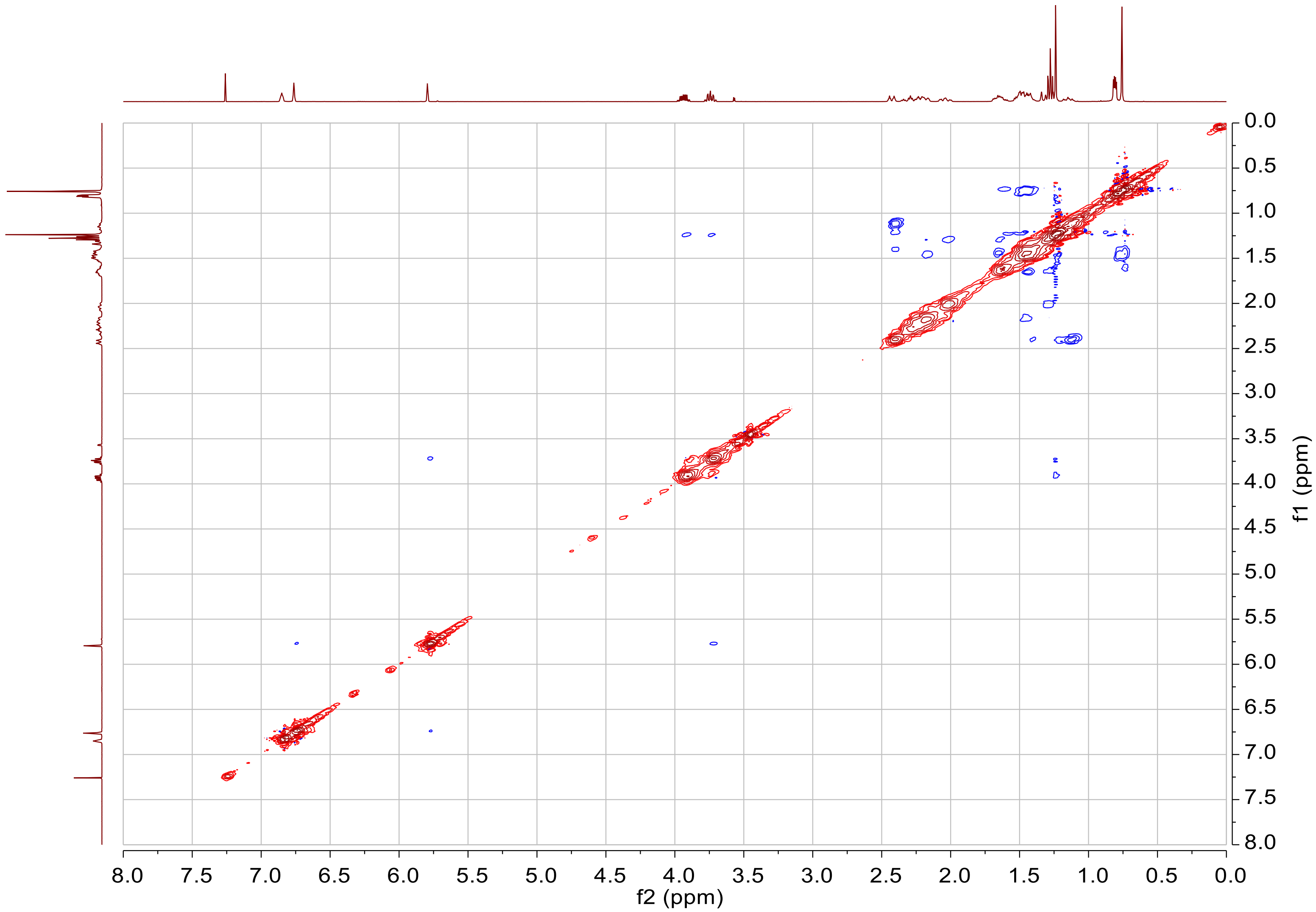

Figure S13 HRESIMS spectrum of callihypolin A (**1**)

Mass Spectrum SmartFormula Report

Analysis Info

Analysis Name	D:\Data\c4\CHF_000004.d	5/7/2019 3:19:23 PM
Method	broadband first signal	Operator: YU HSIAO-CHING
Sample Name	H-F	Instrument: BRUKER FT-MS solariX
Comment	ESI Positive	

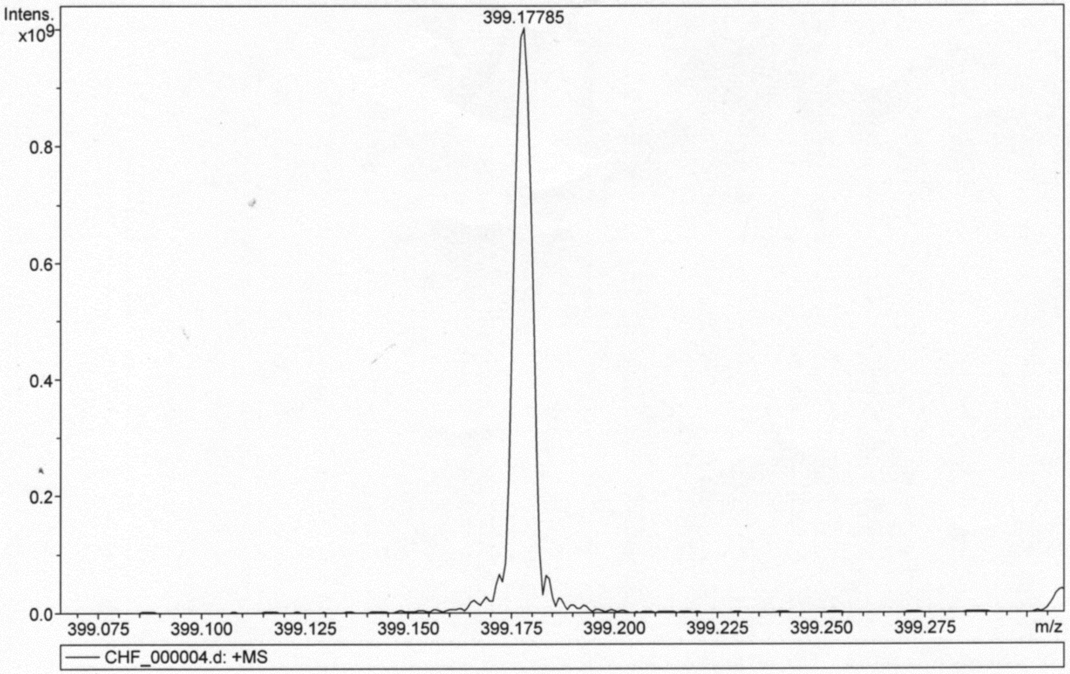

Meas. m/z	#	Formula	Score	m/z	err [mDa]	err [ppm]	mSigma	rdb	e⁻ Conf	N-Rule
399.17785	1	C 21 H 28 Na O 6	100.00	399.17781	-0.04	-0.11	12.1	7.5	even	ok

Figure S14 HRESIMS spectrum of callihypolin B (**2**)

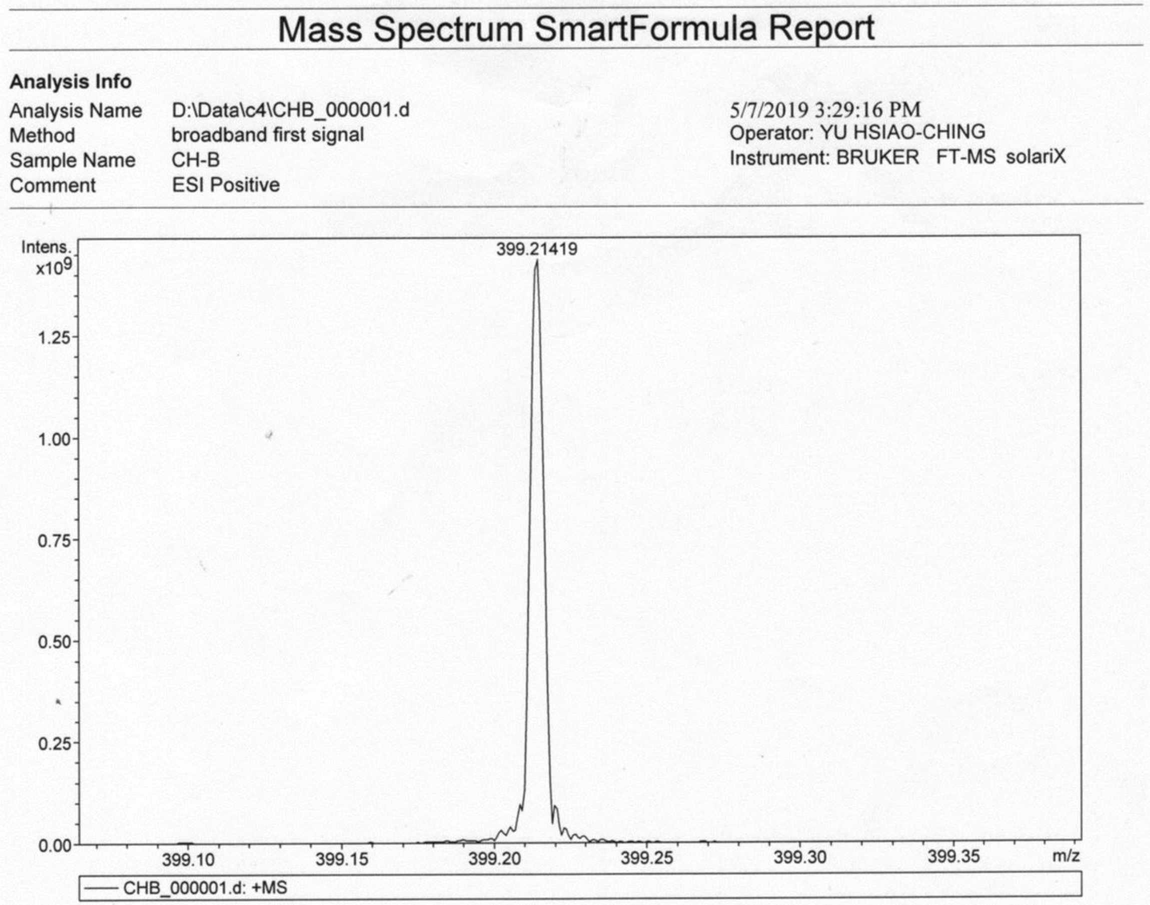

Meas. m/z	#	Formula	Score	m/z	err [mDa]	err [ppm]	mSigma	rdb	e⁻ Conf	N-Rule
399.21419	1	C 22 H 32 Na O 5	100.00	399.21420	0.01	0.02	14.0	6.5	even	ok

Figure S15 Representative traces of superoxide anion generation for compounds **2**–**4**

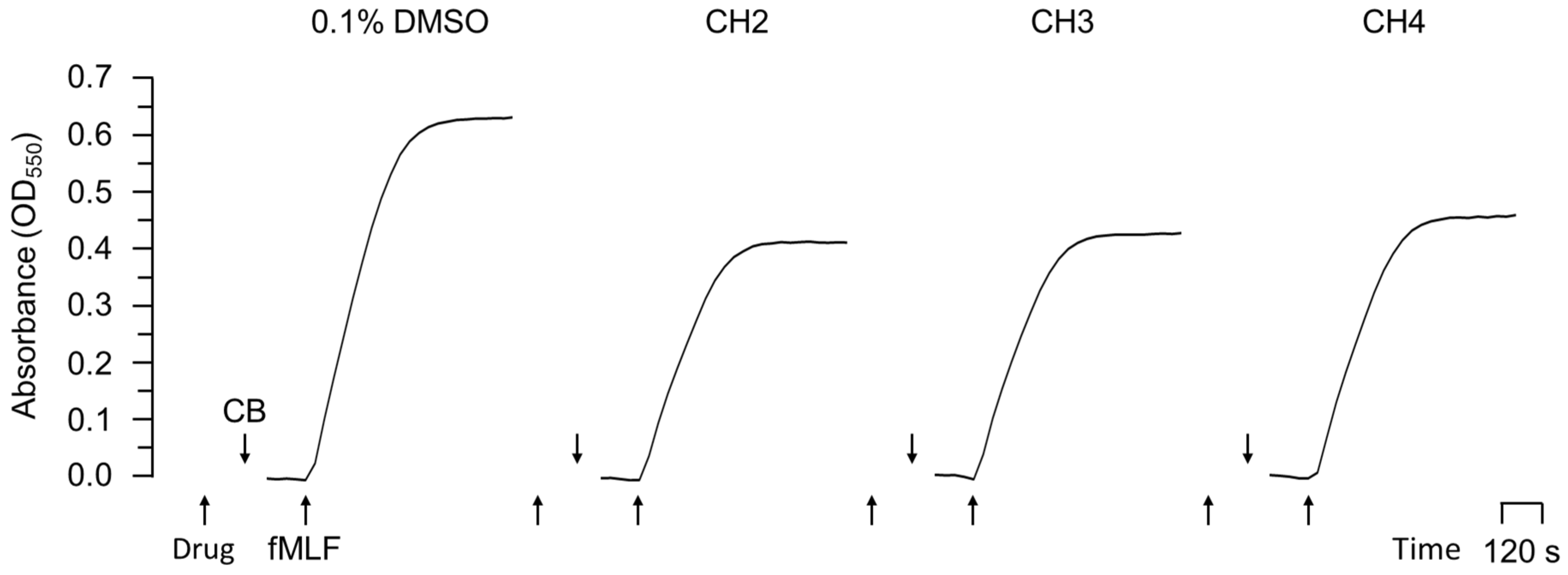

Representative traces are shown for superoxide anion generation.
Compounds: 10 microM

Figure S16 Representative traces of elastase release for compounds **2**–**4**

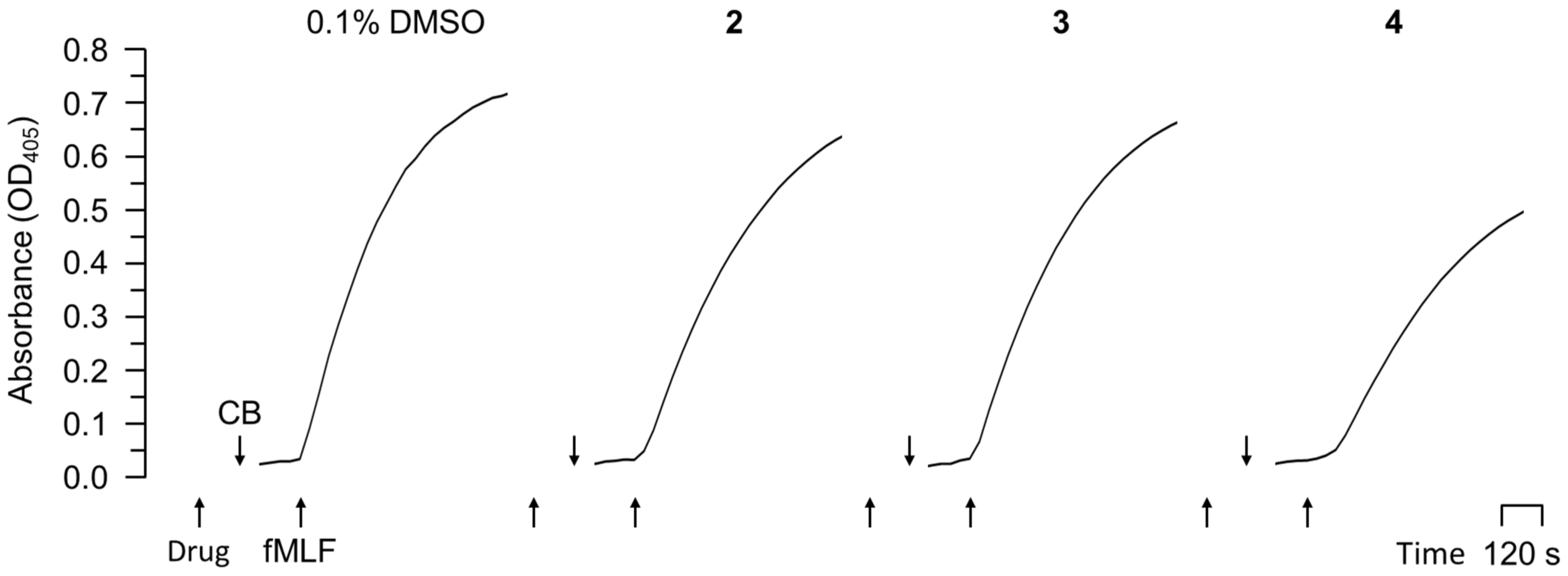

Representative traces are shown for elastase release.
Compounds: 10 microM
